# Heterogeneous Influence of Frailty Phenotypes in Age-Related Hearing Loss and Tinnitus in Chinese Older Adults: An Explorative Study

**DOI:** 10.3389/fpsyg.2020.617610

**Published:** 2021-02-16

**Authors:** Qingwei Ruan, Jie Chen, Ruxin Zhang, Weibin Zhang, Jian Ruan, Min Zhang, Chao Han, Zhuowei Yu

**Affiliations:** ^1^Shanghai Institute of Geriatrics and Gerontology, Shanghai Key Laboratory of Clinical Geriatrics, Huadong Hospital, Research Center of Aging and Medicine, Shanghai Medical College, Fudan University, Shanghai, China; ^2^Department of Otolaryngology, Huadong Hospital, Shanghai Medical College, Fudan University, Shanghai, China; ^3^Department of Geriatrics, Huadong Hospital, Shanghai Medical College, Fudan University, Shanghai, China; ^4^Molecular and Cellular Biology Core Facility, Institute of Neuroscience, Chinese Academy of Sciences, Shanghai, China

**Keywords:** age-related hearing loss, subjective tinnitus, mobility frailty, non-mobility frailty, mobility cognitive frailty, non-mobility cognitive frailty, social dysfunction

## Abstract

**Background:**

Fried physical frailty, with mobility frailty and non-motor frailty phenotypes, is a heterogeneous syndrome. The coexistence of the two phenotypes and cognitive impairment is referred to as cognitive frailty (CF). It remains unknown whether frailty phenotype has a different association with hearing loss (HL) and tinnitus.

**Methods:**

Of the 5,328 community-dwelling older adults, 429 participants aged ≥58 years were enrolled in the study. The participants were divided into robust, mobility, and non-mobility frailty, mobility and non-mobility CF (subdivided into reversible and potentially reversible CF, RCF, and PRCF), and cognitive decline [subdivided into mild cognitive impairment (MCI) and pre-MCI] groups. The severity and presentations of HL and/or tinnitus were used as dependent variables in the multivariate logistic or nominal regression analyses with forward elimination adjusted for frailty phenotype stratifications and other covariates.

**Results:**

Patients with physical frailty (mobility frailty) or who are robust were found to have lower probability of developing severe HL and tinnitus, and presented HL and/or tinnitus than those with only cognitive decline, or CF. Patients with RCF and non-mobility RCF had higher probability with less HL and tinnitus, and the presentation of HL and/or tinnitus than those with PRCF and mobility RCF. Other confounders, age, cognitive and social function, cardiovascular disease, depression, and body mass index, independently mediated the severity of HL and tinnitus, and presented HL and/or tinnitus.

**Conclusion:**

Frailty phenotypes have divergent association with HL and tinnitus. Further research is required to understand the differential mechanisms and the personalized intervention of HL and tinnitus.

**Clinical Trial Registration:**

ClinicalTrials.gov identifier, NCT2017K020.

## Introduction

Frailty is a heterogeneous clinical syndrome with a decline in the functioning of multiple physiological systems, including physical, cognitive, social, or psychosocial frailty phenotypes. It could result in adverse outcomes, such as dependency, falls, disability, and death (Andrew et al., 2008; Ruan et al., 2015; Bunt et al., 2017; Hoogendijk et al., 2019). The coexistence of physical frailty and cognitive impairment in older adults is defined as cognitive frailty (CF) (Kelaiditi et al., 2013), including reversible cognitive frailty (RCF) and potential reversible cognitive frailty (PRCF) based on the severity of cognitive impairment (Ruan et al., 2015). Physical frailty could be divided into mobility and non-mobility frailty phenotypes (Liu et al., 2017). Motor dysfunctions, such as slowness and/or weakness, are the important components of physical frailty and CF (Fried et al., 2001; Kelaiditi et al., 2013; Ruan et al., 2015). Individuals with pre-frailty phenotype (one or two of weakness, slowness, and low physical activity) had a faster development trajectory of adverse outcomes than those with exhaustion and/or unexplained weight loss (Romero-Ortuno et al., 2019). Slowness and/or weakness were defined as the core of mobility frailty phenotype (Liu et al., 2017) and are closely associated with cognitive impairment (Boyle et al., 2009; Mielke et al., 2013). The coexistence of mobility or non-mobility frailty with cognitive decline may be referred to as mobility or non-mobility CF. The simultaneous presence of gait disturbances and cognitive decline, as a phenotype of mobility CF, was also defined as motoric cognitive risk syndrome, which has been proposed as a new powerful predictor of dementia and age-related adverse outcomes (Chhetri et al., 2017). Social frailty, social vulnerability, or social dysfunction also increased the risk of adverse outcomes such as fitness and mortality (Andrew et al., 2008), age-related hearing loss (HL), cognitive deficits, depression, and tinnitus in older adults (Li et al., 2015; Panza et al., 2015; Lozupone et al., 2018; Ma et al., 2018; Yoo et al., 2019; Loughrey et al., 2020).

Hearing loss, or presbycusis, which is the most common sensory dysfunction, is an important component of frailty index, and both presbycusis and the degree of frailty index are associated with a higher risk of developing cognitive impairment and dementia (Panza et al., 2015; Deal et al., 2017; Wallace et al., 2019). The coexistence of physical frailty and HL in older adults was related to a worse cognitive performance compared with HL alone (Bonfiglio et al., 2020). Furthermore, cognitive impairment and depressive symptoms may be present during subclinical HL (Golub et al., 2019, 2020). The occurrence of HL on at least one side in older adults can significantly affect the motor functions and increase the risk of postural instability and falls (Bang et al., 2020); it is independently associated with mobility frailty (Kamil et al., 2016), greater disability, and limitations in multiple self-reported difficulties in physical functioning (Chen et al., 2014). HL could result in loneliness and social isolation due to communication difficulty. Social factors might mediate the association between HL and episodic memory (Loughrey et al., 2020). HL in combination with low social activity was an independent risk factor of the development of a disability (Bae et al., 2018). Improving the social networks of older adults with HL by intervention could decrease HL-associated episodic memory impairment (Maharani et al., 2019).

Subjective tinnitus is another common comorbid disorder of the auditory system in older adults. Apart from an aberrant auditory sensory perception, chronic tinnitus is closely associated with cognitive deficits and emotional, psychological, and mental health disorders, such as depression, anxiety, and sleep disturbance or insomnia (Langguth et al., 2013; Ruan et al., 2018; Jafari et al., 2019). Chronic tinnitus-related cognitive impairment includes working memory, executive control of attention, and processing speeds. Higher physical activity in individuals with tinnitus had lower levels of tinnitus severity (Carpenter-Thompson et al., 2015). However, studies on the association between chronic tinnitus and other components of physical frailty are extremely scarce. The contribution of tinnitus and/or HL to the development of cognitive decline and CF subtypes in older adults is not well understood. As sensory and motor regions of the central nervous system are affected by Alzheimer’s disease (AD) pathology (Albers et al., 2015; Maharani et al., 2019; Loughrey et al., 2020), we hypothesized that patients with a frailty phenotype that involves cognitive or mobility decline had higher risks of severe HL and tinnitus, and presented HL and/or tinnitus.

Hence, the present study aimed to investigate the association between the severity of age-related HL or chronic tinnitus and frailty phenotypes, including CF subtypes, as well as the association between the presentation of HL and/or tinnitus with frailty phenotype stratifications.

## Methods

### Design and Setting

The participants of the Shanghai study of health promotion for elderly individuals with frailty, which is a population-based cross-sectional study, were enrolled in the present study (Ruan et al., 2020c). We analyzed the demographic, health, social, and neuropsychological data of individuals aged 58 years and above.

### Participants

After excluding individuals with severe disability, complete loss of hearing and vision, and dementia based on the Diagnostic and Statistical Manual of Mental Disorders, Fourth Edition (American Psychiatric Association, 1994), 429 qualified volunteers were recruited from the previous cohort (Ruan et al., 2020c). The individuals were divided into robust, mobility and non-mobility frailty, mobility and non-mobility CF (including RCF and PRCF), and cognitive decline (including pre-MCI and MCI) (Table 1).

**TABLE 1:** Sample characteristic (medians and interquartile ranges [Q25–Q75] for continuous variables, and absolute numbers or percentages for categorical variables) of 429.

**Variable**	**Full sample (*n* = 429)**	**The status of frailty phenotypes**									***P*-value**
		**Robust (*n* = 105; 24.5%)**	**Mobility frailty (*n* = 68; 15.9%)**	**Non-mobility frailty (*n* = 40; 9.30%)**	**Mobility CF (*n* = 107, 24.94%)**		**Non-mobility CF (*n* = 31, 7.23%)**		**Only cognitive impairment (*n* = 78, 18.18%)**		
					**Mobility RCF (*n* = 51; 11.90%)**	**Mobility PRCF (*n* = 56; 13.10%)**	**Non-mobility RCF (*n* = 19; 4.40%)**	**Non-mobility PRCF (*n* = 12; 2.80%)**	**Only pre-MCI (*n* = 43; 10.00%)**	**Only MCI (*n* = 35; 8.20%)**	
**Demographics**											
Age (mean ± SD)	72.00 (67.00, 78.00)	70.00 (65.00, 75.50)	76.00 (70.25, 81.75)	68.00 (64.25, 73.00)	75.00 (69.00, 79.00)	76.50 (72.00, 81.00)	76.00 (68.00, 80.00)	73.00 (67.50, 77.50)	70.00 (65.00, 75.00)	72.00 (67.00, 76.00)	0.000
Female (n;%)	246 (57.30)	55(52.40)	32 (47.10)	24 (60.00)	34 (66.70)	32 (57.10)	14 (73.70)	6 (50.00)	26 (60.50)	23 (65.70)	0.307
Education	12.00 (9.00, 15.00)	12.00 (9.00, 15.00)	14.00 (10.00, 16.00)	12.00 (9.00, 15.00)	9.00 (9.00, 15.00)	9.00 (8.00, 12.00)	11.00 (9.00, 12.00)	11.50 (9.00, 16.00)	12.00 (9.00, 15.00)	9.00 (9.00, 12.00)	0.000
**HL category**	425	104 (24.47)	68 (16.00)	40 (9.41)	51 (12.00)	56 (13.18)	19 (4.47)	10 (2.35)	42 (9.88)	35 (8.24)	0.000
0	184 (43.30)	60 (57.69)	39 (57.35)	21 (52.50)	15 (29.41)	7 (12.50)	10 (52.63)	2 (20.00)	23 (54.76)	7 (20.00)	
1	129 (30.40)	30 (28.85)	13 (19.12)	12 (30.00)	20 (39.22)	20 (35.71)	8 (42.11)	1 (10.00)	10 (23.81)	15 (42.86)	
2	112 (26.40)	14 (13.46)	16 (23.53)	7 (17.50)	16 (31.37)	29 (51.79)	1 (5.26)	7 (70.00)	9 (21.43)	13 (37.14)	
**THI score**	427	105 (24.59)	67 (15.69)	40 (9.37)	50 (11.71)	56 (13.11)	19 (4.45)	12 (2.81)	43 (10.07)	35 (8.20)	0.177
0	279 (65.30)	77 (73.33)	48 (71.64)	24 (60.00)	32 (64.00)	30 (53.57)	13 (68.42)	7 (58.33)	26 (60.47)	22 (62.86)	
1	61 (14.30)	21 (20.00)	16 (23.88)	9 (22.50)	13 (26.00)	15 (26.79)	5 (26.32)	5 (41.67)	13 (30.23)	11 (31.43)	
2	24 (5.60)	7 (6.67)	3 (4.48)	7 (17.50)	5 (10.00)	11 (19.64)	1 (5.26)	0 (0.00)	4 (9.30)	2 (5.71)	
**HL and tinnitus**	424	104 (24.53)	67 (15.80)	40 (9.43)	51 (12.03)	56 (13.21)	19 (4.48)	10 (2.36)	42 (9.91)	35 (8.25)	0.003
0	113 (26.70)	36 (34.62)	24 (35.82)	13 (32.50)	7 (13.73)	6 (10.71)	7 (36.84)	2 (20.00)	14 (33.33)	4 (11.43)	
1	105 (24.80)	23 (22.12)	15 (22.39)	8 (20.00)	15 (29.41)	20 (35.71)	2 (10.53)	4 (40.00)	7 (16.67)	11 (31.43)	
2	68 (16.00)	22 (21.15)	14 (20.90)	8 (20.00)	8 (15.69)	1 (1.79)	3 (15.79)	0 (0.00)	9 (21.43)	3 (8.57)	
3	138 (32.50)	23 (22.12)	14 (20.90)	11 (27.50)	21 (41.18)	29 (51.79)	7 (36.84)	4 (40.00)	12 (28.57)	17 (48.57)	
**Covariates**											
BMI (*n* = 421)	24.40 (22.00, 26.20)	24.10 (21.95, 26.40)	24.60 (21.60, 26.00)	24.90 (22.43, 25.78)	24.90 (22.00, 26.40)	25.05 (23.08, 27.30)	23.80 (20.80, 25.80)	22.60 (19.98, 25.60)	24.40 (22.60, 25.90)	24.30 (21.35, 26.00)	0.334
Chronic comorbility	398	95	66	35	48	55	18	11	38	32	0.003
0	49 (12.30)	18 (18.90)	10 (15.20)	3 (8.60)	3 (6.30)	3 (5.50)	1 (5.60)	2 (18.20)	4 (10.50)	5 (15.60)	
1	127 (31.90)	39 (41.10)	15 (22.70)	10 (28.60)	12 (25.00)	14 (25.50)	4 (22.20)	0 (0.0.00	17 (44.70)	16 (50.00)	
2	124 (31.20)	26 (27.40)	23 (34.80)	11 (31.40)	19 (39.60)	15 (27.30)	7 (38.90)	6 (54.50)	12 (31.60)	5 (15.60)	
≥ 3	98 (24.60)	12 (12.60)	18 (27.30)	11 (31.40)	14 (29.20)	23 (41.80)	6 (33.30)	3 (27.30)	5 (13.20)	6 (18.80)	
CVD	254/428 (59.30)	53 (50.50)	39 (58.20)	18 (45.00)	37 (72.50)	38 (67.90)	13 (68.40)	8 (66.70)	27 (62.80)	21 (60.00)	0.100
diabetes mellitus	76/428 (17.80)	6 (5.70)	12 (17.90)	5 (12.50)	14 (27.50)	19 (33.90)	2 (10.50)	9 (25.00)	9 (20.90)	6 (17.10)	0.001
Stroke	42/428 (9.80)	5 (4.80)	6 (9.00)	6 (15.00)	6 (11.80)	15 (26.80)	0 (0.00)	2 (16.70)	2 (4.70)	0 (0.00)	0.000
non-skin malignancy	31/428 (7.20)	6 (5.70)	7 (10.40)	3 (7.50)	2 (3.90)	5 (8.90)	1 (5.30)	1 (8.30)	1 (2.30)	5 (14.30)	0.566
Social dysfunction	28.00 (24.00, 26.20)	26.00 (23.00, 29.75)	27.00 (25.00, 33.50)	29.00 (27.00, 42.75)	29.00 (24.00, 38.25)	31.00 (25.00, 38.50)	33.00 (26.00, 34.50)	35.00 (28.00, 42.00)	25.50 (22.00, 32.00)	27.50 (23.25, 33.00)	0.001
MMSE	419 (26.00, 29.00)	99 (27.00, 29.00)	67 (27.00, 29.00)	39 (27.00, 29.00)	51 (26.00, 28.00)	55 (25.00, 28.00)	19 (25.00, 28.00)	12 (27.00, 29.00)	42 (27.00, 29.00)	35 (25.00, 28.00)	0.000
GDS 15	420	104	65	40	50	55	19	10	42	35	0.328
<6	317 (75.48)	84 (80.80)	49 (75.40)	25 (62.50)	34 (68.00)	40 (72.70)	15 (78.90)	8 (80.00)	32 (76.20)	30 (75.50)	
≥6	103 (24.52)	20 (19.20)	16 (24.60)	15 (37.50)	16 (32.00)	15 (27.30)	4 (21.10)	2 (20.00)	10 (23.80)	5 (14.30)	
Smoking status	422	103	68	38	51	56	19	11	42	34	0.317
Never	353 (83.60)	83 (80.60)	60 (88.20)	26 (68.40)	46 (90.20)	48 (85.70)	16 (84.00)	10 (90.90)	38 (90.50)	26 (76.50)	
Previous	38 (9.00)	11 (10.70)	6 (8.80)	6 (15.80)	3 (5.90)	6 (10.70)	1 (5.30)	0 (0.00	2 (4.80)	3 (8.80)	
Current	31 (7.30)	9 (8.70)	2 (2.90)	6 (15.80)	2 (3.90)	2 (3.60)	2 (10.50)	1 (9.10)	2 (4.80)	5 (14.70)	
Alcohol intake	422	104	68	37	51	56	19	11	42	34	0.561
Never	378 (89.60)	90 (86.50)	62 (91.20)	31 (83.80)	46 (90.20)	52 (92.90)	17 (89.50)	10 (90.90)	40 (95.20)	30 (88.20)	
Previous	14 (3.30)	7 (6.70)	0 (0.00)	1 (2.70)	1 (2.00)	1 (1.80)	1 (5.30)	1 (9.10)	0 (0.00)	2 (5.90)	
Current	30 (7.10)	7 (6.70)	6 (8.80)	5 (13.50)	4 (7.80)	3 (5.40)	1 (5.30)	0 (0.00)	2 (4.80)	2 (5.90)	
TFI (*n* = 426)	3.00 (0.00, 33.30)	0.00 (0.00, 26.60)	0.00 (0.00, 22.80)	0.00 (0.00, 49.90)	17.20 (0.00, 39.10)	18.40 (0.00, 40.80)	8.00 (0.00, 41.00)	0.00 (0.00, 31.00)	2.80 (0.00, 26.80)	14.00 (0.00, 40.00)	0.385
**Neuropsychological test Z-scores**											
TMT A (*n* = 416)	−0.13 (−0.56, 0.71)	−0.38 (−0.73, −0.06)	−0.38 (−0.66, 0.10)	−0.20 (−0.59, 0.05)	−0.05 (−0.33, 1.02)	0.78 (−0.10, 2.49)	0.72 (−0.56, 1.55)	0.85 (−0.09, 3.59)	0.01 (−0.47, 0.71)	1.24 (0.49, 1.99)	0.000
TMT B (*n* = 415)	−0.09 (−0.56, 0.46)	−0.37 (−0.65, −0.02)	−0.18 (−0.70, 0.19)	−0.18 (−0.69, 0.03)	−0.10 (−0.56, 0.45)	0.61 (−0.28, 1.77)	0.002 (−0.61, 0.40)	1.29 (−0.01, 1.81)	−0.06 (−0.48, 0.17)	0.96 (−0.11, 1.77)	0.000
Delay recall (*n* = 420)	−0.30 (−0.97, 0.44)	0.29 (−0.30, 0.89)	0.03 (−0.32, 0.70)	−0.22 (−0.51, 0.46)	−0.52 (−1.08, 0.20)	−1.10 (−1.47, −0.25)	−0.82 (−1.32, −0.23)	−0.90 (−1.49, −0.46)	−0.79 (−1.51, −0.47)	−0.90 (−1.61, −0.29)	0.000
Recognition (*n* = 421)	0.06 (−0.65, 0.53)	0.29 (−0.27, 0.71)	0.50 (−0.04, 0.87)	0.11 (−0.57, 0.69)	−0.15 (−0.64, 0.42)	−0.51 (−1.74, 0.18)	0.06 (−0.47, 0.40)	−0.89 (−1.75, 0.55)	−0.51 (−1.14, 0.32)	−0.46 (−1.01, 0.11)	0.000
Learning slope (*n* = 421)	−0.03 (−0.59, 0.51)	0.23 (−0.28, 0.79)	0.21 (−0.26, 0.75)	0.25 (−0.25, 0.80)	−0.24 (−1.34, 0.46)	−0.23 (−0.70, 0.46)	−0.51 (−0.94, 0.49)	−0.75 (−1.07, 0.11)	−0.46 (−1.20, 0.03)	−0.28 (−1.02, 0.45)	0.000
Intrusion errors (*n* = 421)	−0.24 (−0.77, 0.49)	−0.34 (−0.65, 0.40)	−0.28 (−0.78, 0.18)	−0.48 (−1.04, −0.11)	0.16 (−0.75, 0.99)	−0.46 (−1.03, 0.65)	0.12 (−0.55, 0.92)	−0.09 (−0.38, 0.50)	0.17 (−0.77, 1.31)	−0.28 (−0.77, 0.49)	0.045
Retroactive interference (*n* = 421)	−0.11 (−0.61, 0.39)	0.07 (−0.28, 0.55)	0.12 (−0.55, 0.62)	0.14 (−0.28, 0.53)	−0.23 (−0.90, 0.14)	−0.22 (−1.18, 0.30)	−0.47 (−1.20, −0.27)	−0.15 (−0.89, 0.25)	−0.19 (−0.98, 0.47)	−0.24 (−1.04, 0.12)	0.000
BNT (*n* = 422)	−0.12 (−0.78, 0.37)	0.26 (−0.29, 0.68)	0.10 (−0.29, 0.46)	0.06 (−0.29, 0.50)	−0.26 (−0.98, 0.33)	−1.20 (−1.63, −0.49)	−0.28 (−1.43, 0.16)	−0.12 (−0.95, 0.05)	−0.07 (−0.74, 0.16)	−0.88 (−1.22, −0.02)	0.000
Animal fluency (*n* = 422)	−0.33 (−0.87, 0.35)	0.10 (−0.36, 0.88)	−0.09 (−0.67, 0.60)	0.13 (−0.44, 0.52)	−0.77 (−1.19, −0.17)	−1.04 (−1.45, −0.42)	−0.46 (−0.80, 0.22)	−0.46 (−1.26, −0.13)	−0.42 (−0.84, 0.32)	−0.63 (−1.26, −0.35)	0.000

*HL, hearing loss; BMI, body mass index; CVD, cardiovascular disease; GDS, the Geriatric Depression Scale; THI, handicap inventory; TFI, tinnitus functional index; TMT A and B, Trail Making Test A and B; BNT, Boston naming; CF, cognitive frailty; RCF, reversible cognitive frailty; PRCF, potential reversible cognitive frailty; MCI, mild cognitive impairment. HL 0 = Normal hearing; HL 1 = mild HL; and HL 3 = moderate or severe HL. Tinnitus 0 = no tinnitus; tinnitus 1 = mild or moderate tinnitus; tinnitus 2 = severe or disastrous tinnitus. HL and tinnitus 0 = without HL and tinnitus; HL and tinnitus 1 = only HL; HL and tinnitus 0 = only tinnitus; HL and tinnitus 3 = with HL and tinnitus.*

### Ethical Approval

The study was approved by the Ethics Committee of Huadong Hospital (Approval No. Ref 2018K055), and written informed consent was obtained from each volunteer or authorized representative.

### Measurements

Hearing was objectively measured using a pure-tone audiometry in a sound-attenuating booth according to the American National Standards Institute standards. The air conduction thresholds in each ear [in decibel (dB) hearing level] were measured from 500 to 8,000 Hz. The pure-tone average (PTA) in the better hearing ear was calculated using the 0.5-, 1-, 2-, and 4-kHz thresholds (World Health Organization, 2015). The participants were divided into groups based on the hearing levels: normal hearing (PTA ≤ 25 dB), mild loss (>25 and ≤40 dB), moderate loss (>40 and ≤70 dB), and severe loss (>70 dB) (Lin et al., 2013).

The Tinnitus Handicap Inventory (THI) is validated in Chinese people with a 25-item self-rating instrument and can yield a score (0, 2, or 4, which correspond to “not affected,” “sometimes affected,” and “always affected,” respectively) from 0 to 100. THI includes items concerning general tinnitus severity, quality of life, and psychological aspects of tinnitus (Newman et al., 1996). Tinnitus severity is divided into three levels, including the mild (1–16 and 18–36), the moderate (38–56 and 58–76), and the disaster (78–100) level according to the THI scores (1–100). The Tinnitus Functional Index (TFI) is a standardized tinnitus severity assessment tool, and its score is the total scores of eight domains including intrusive, sense of control, cognitive, sleep, auditory, relaxation, emotional, and quality of life (Carpenter-Thompson et al., 2015). All individuals with self-reported chronic subjective tinnitus (more than 3 months) were required to complete the THI and TFI.

The objective assessment of cognitive performance had been reported in the literature (Thomas et al., 2018, 2020). In the present study, the MCI and pre-MCI evaluation were conducted using normative z-scores of neuropsychological test battery, including Trail Making Test A and B (TMT A and B) for executive or attention domain; Boston Naming Test (BNT) and Animal List generation for language domain; the Hopkins Verbal Learning Test-Revised (HVLT-R) for memory domain, including delayed free correct responses and HVLT-R recognition; and three process scores from the HVLT-R (Ruan et al., 2020b). Two impaired process scores, one impaired process score and one impaired total score, impaired total score on two measures across different cognitive domains or a Functional Assessment Questionnaire (FAQ) score of 6–8 was classified as pre-MCI. Impaired total score on two measures in the same domain, one impaired score in each of the three cognitive domains or a FAQ score of ≥9 was classified as MCI (Thomas et al., 2018, 2020).

The mobility and non-mobility frailty phenotypes were evaluated using the five-item Fried scale with Chinese reference values (Hao et al., 2017). Mobility frailty was marked by weakness and/or slowness, whereas non-mobility frailty was indicated by the existence of at least one of the following criteria: unexplained weight loss, fatigue, and low physical activity after excluding mobility frailty (Liu et al., 2017). The CF groups were further divided into mobility or non-mobility RCF if the individuals had both mobility or non-mobility frailty and pre-MCI and mobility or non-mobility PRCF if the individuals had both mobility or non-mobility frailty and MCI.

Demographic information (including age, sex, and education level), self-reported smoking, alcohol intake, and chronic comorbidity, which were validated by conducting a medical chart review were obtained by trained medical staff in 2018–2019. Chronic comorbidity was evaluated according to our previous study (Ruan et al., 2020a). A total of 13 chronic disorders were included: diabetes mellitus, cardiovascular disease, osteoporosis, stroke, arthritis, chronic obstructive lung disease, anemia, peripheral vascular disease, Alzheimer’s disease, Parkinson’s disease, mental or psychiatric disorders, chronic renal disease, and non-skin malignancy. Cardiovascular disease (CVD) includes coronary problems (myocardial infarction/heart attack or angina pectoris), hypertension, congestive heart failure, or cardiac arrhythmia. Global cognitive status was evaluated by using a Mini Mental Status Examination (MMSE). Depression was assessed by using the 15-item short form of the Geriatric Depression Scale (GDS) (Chau et al., 2006). Social dysfunction was assessed by using the 21-item Social Dysfunction Rating Scale (Linn et al., 1969). Body mass index (BMI) was calculated as weight in kilograms divided by height in meter squared.

### Statistical Analysis

The distribution of continuous variables of frailty phenotype stratifications was tested for normality using the Kolmogorov–Smirnov Test. The difference between the groups was analyzed using a bivariate correlation (Pearson’s test for normally distributed variables or Spearman’s test for variables with non-normal distribution). The difference in categorical variables among groups was tested *via* one-way analysis of variance. If the test of homogeneity of variances was inappropriate, the Mann–Whitney *U*-test was employed to analyze the univariate correlation. Some categorical data were expressed as a proportion and compared using the χ^2^ test. All significant categorical and continuous variables associated with HL and/or tinnitus were further analyzed using multivariate logistic regression or nominal regression with forward elimination. The *p*-value for multiple comparisons was corrected, and a *p*-value < 0.05 was considered significant. All analyses were conducted using the SPSS 18.0 software.

## Results

Table 1 presents the characteristics of robust; mobility frailty; non-mobility frailty; mobility CF, including mobility RCF and mobility PRCF; non-mobility CF, including non-mobility RCF and non-mobility PRCF; and cognitive decline, including pre-MCI and MCI patients. The distribution of HL (*p* < 0.001) and the presentation of HL and/or tinnitus (*p* = 0.003) were significantly different among frailty phenotype stratifications. Tinnitus severity based on THI score (*p* = 0.177) and TFI score (*p* = 0.385) was not significantly different among frailty phenotype stratifications (Table 1).

After the adjustment for confounders, the robust and mobility frailty phenotypes were associated with significantly higher odds of normal hearing [odds ratio (OR), 5.99 and 6.82, respectively] than that of moderate and severe HL when compared with the cognitive decline phenotype (model 3, Table 2). When compared with the MCI group, the mobility frailty was associated with significantly higher odds (OR, 12.69) of normal hearing than that of the moderate and severe HL (model 4, Table 2). After dividing CF into RCF and PRCF, and cognitive decline being divided into pre-MCI and MCI, the mobility RCF (OR, 3.28; *p* = 0.076), non-mobility RCF (OR, 27.43), and pre-MCI (OR, 4.06) phenotypes were associated with higher odds of normal hearing, and the non-mobility RCF group with higher odds of mild HL (OR, 8.33, *p* = 0.065) than of the moderate and severe HL (model 5, Table 2) when compared with the MCI phenotype. After excluding non-mobility frailty phenotypes in model 6 of Table 2, including non-mobility frailty, non-mobility RCF, and PRCF, the robust (OR, 9.11) and mobility frailty (OR, 11.32) phenotypes were associated with significantly higher odds of normal hearing. After the adjustment for covariates, age was an independent risk factor of the severity of HL among different frailty stratifications in all six models (Table 2). TMT B and animal fluency scores (OR, 0.62, *p* = 0.06) in model 3 and TMT B in model 4 were also independent risk factors of the severity of HL.

**TABLE 2:** Association between frailty phenotype and the severity of hearing loss by using multivariate logistic regression or nominal regression.

	**HL (0, ref: 2)**					
	**Model 1 OR (95%CI)**	**Model 2 OR (95%CI)**	**Model 3 OR (95%CI)**	**Model 4 OR (95%CI)**	**Model 5 OR (95%CI)**	**Model 6 OR (95%CI)**
**Frailty phenotypes**						
Robust	NA	**4.04 (0.87, 18.76)**	5.99 (1.38, 25.95)*	NA	NA	9.11 (1.40, 59.18)*
Mobility frailty	NA	NA	6.82 (1.48, 31.50)*	12.69 (1.95, 82.55)**	NA	11.32 (1.61, 79.51)*
Non-mobility frailty	NA	NA	2.88 (0.41, 20.34)	5.45 (0.60, 49.65)	NA	NA
Mobility CF	0.53 (0.21, 1.32)	0.90 (0.24, 3.35)	0.95 (0.31, 2.95)	1.79 (0.39, 8.27)	NA	NA
Mobility RCF	NA	NA	NA	NA	**3.28 (0.88, 12.19)**	2.60 (0.43, 15.72)
Mobility PRCF	NA	NA	NA	NA	0.66 (0.17, 2.56)	0.47 (0.06, 3.52)
Non-mobility CF	3.13 (0.73, 13.38)	4.40 (0.33, 59.10)	2.57 (0.44, 15.21)	4.77 (0.61, 37.17)	NA	NA
Non-mobility RCF	NA	NA	NA	NA	27.43 (2.50, 301.48)**	NA
Non-mobility PRCF	NA	NA	NA	NA	0.93 (0.13, 6.88)	NA
Cognitive decline	0^a^	0^a^	0^a^	–	–	–
Pre-MCI	NA	NA	NA	3.43 (0.51, 23.23)	4.06 (1.04, 15.90)*	2.38 (0.32, 17.65)
MCI	–	–	–	0^a^	0^a^	0^a^
Age	0.85 (0.79, 0.91)***	0.84 (0.76, 0.92)***	0.87 (0.81, 0.93)***	0.87 (0.81, 0.94)***	0.85 (0.80, 0.91)***	0.84 (0.77, 0.92)***
GDS15	–	0.79 (0.65, 0.96)*	–	–	–	–
TMT B	–	–	0.784 (0.53, 1.15)	0.79 (0.52, 1.17)	–	–
Animal fluency	–	–	1.05 (0.64, 1.72)	–	–	–
	HL (1, ref: 2)					
**Frailty phenotypes**						
Robust	NA	2.55 (0.58, 11.17)	2.42 (0.61, 9.65)	NA	NA	**4.65 (0.82, 26.29)**
Mobility frailty	NA	NA	0.61 (0.12, 3.12)	0.49 (0.08, 2.80)	NA	1.55 (0.22, 10.91)
Non-mobility frailty	NA	NA	1.06 (0.14, 7.84)	0.81 (0.10, 6.49)	NA	NA
Mobility CF	0.88 (0.38,2.01)	0.66 (0.21, 2.11)	**0.39 (0.14, 1.04)**	0.43 (0.14, 1.32)	NA	NA
Mobility RCF	NA	NA	NA	NA	1.36 (0.45, 4.09)	1.35 (0.27, 6.90)
Mobility PRCF	NA	NA	NA	NA	0.65 (0.23, 1.82)	0.62 (0.12, 3.23)
Non-mobility CF	2.43(0.61, 9.62)	2.76 (0.23, 32.63)	0.81 (0.14, 4.60)	0.85 (0.14, 5.15)	NA	NA
Non-mobility RCF	NA	NA	NA	NA	**8.33 (0.88, 79.18)**	NA
Non-mobility PRCF	NA	NA	NA	NA	0.18 (0.02, 1.80)	NA
Cognitive decline	0^a^	0^a^	0^a^	–	–	–
Pre-MCI	NA	NA	NA	0.76 (0.15, 3.82)	1.06 (0.29, 3.81)	1.88 (0.30, 11.77)
MCI	–	–	–	0^a^	0^a^	0^a^
Age	0.96 (0.90, 1.01)	0.96 (0.89, 1.05)	0.98 (0.92, 1.04)	0.97 (0.91, 1.03)	0.96 (0.91, 1.02)	0.95 (0.88, 1.03)
GDS15	–	0.87(0.73, 1.04)	–	–	–	–
TMT B	–	–	0.60 (0.42, 0.86)**	0.61 (0.42, 0.89)**	–	–
Animal fluency	–	–	0.62 (0.38, 1.02)	–	–	–

^*a*^means reference category; OR = odds ratios; CI = confidence intervals; NA, not applicable; CF = cognitive frailty; RCF = reversible cognitive frailty; PRCF = potential reversible cognitive frailty; MCI, mild cognitive impairment; BMI, body mass index; HL, hearing loss; TMT B, Trail Making Test B; GDS, the Geriatric Depression Scale. Model 1 is adjusted for mobility CF, non-mobility CF, cognitive decline, and age. Model 2 is adjusted for robust, mobility CF, non-mobility CF, cognitive decline, age, and GDS15. Model 3 is adjusted for robust, mobility frailty; non-mobility frailty; mobility CF, non-mobility CF, cognitive decline, age, TMT B, and animal fluency. Model 4 is adjusted for mobility frailty; non-mobility frailty; mobility CF, non-mobility CF, pre-MCI, MCI, age, and TMT B. Model 5 is adjusted for mobility RCF, mobility PRCF, non-mobility RCF, non-mobility PRCF, pre-MCI, MCI, and age. Model 6 is adjusted for robust, mobility frailty; mobility RCF, mobility PRCF, pre-MCI, MCI, and age. HL 0 = Normal hearing; HL 1 = mild HL; and HL 3 = moderate or severe HL. Chinese adults aged 58 years and older and stratified by frailty phenotypes. * *p* < 0.05; ** *p* ≤ 0.01; and *** *p* < 0.001; bold values denote marginally statistical significance.

Compared with the cognitive decline or MCI group, other frailty stratifications were not associated with odds of without tinnitus or with severe and disastrous tinnitus (models 1–6, Table 3); however, the mobility CF (OR, 0.36, 0.24, 0.26, and 0.20; *p* = 0.066, 0.086, 0.075, and 0.07 in models 1–4) or mobility RCF phenotype (OR, 0.21; *p* = 0.074 in model 5) was associated with marginally lower odds of mild and moderate tinnitus than that of severe and disastrous tinnitus. BMI was an independent factor associated with the tinnitus severity in all three groups in model 1 of Table 3. Among the four stratifications in model 2 of Table 3, patients experiencing depression were associated with higher odds of severe and disastrous tinnitus than those without tinnitus (OR, 0.83). Patients with social dysfunction were associated with higher odds of severe and disastrous tinnitus than those without tinnitus (model 3, Table 3). Other confounders, CVD (OR, 0.19; model 3), the z-scores of TMT A (OR, 0.75, 0.75, and 0.80; models 3–5), recognition (OR, 3.74), and Boston naming scores (OR, 2.88; *p* = 0.054; model 6) were independently associated with the severity of tinnitus.

**TABLE 3:** Association between frailty phenotype and the severity of tinnitus by using multivariate logistic regression or nominal regression.

	**Tinnitus (0, ref: 2)**					
	**Model 1 OR (95%CI)**	**Model 2 OR (95%CI)**	**Model 3 OR (95%CI)**	**Model 4 OR (95%CI)**	**Model 5 OR (95%CI)**	**Model 6 OR (95%CI)**
**Frailty phenotypes**						
Robust	NA	0.58 (0.16, 2.10)	0.80 (0.24, 2.66)	NA	NA	0.84 (0.14, 5.01)
Mobility frailty	NA	NA	1.72 (0.38, 7.90)	1.71 (0.32, 9.08)	NA	1.65 (0.21, 13.09)
Non-mobility frailty	NA	NA	1.21 (0.17, 8.57)	0.84 (0.12, 6.01)	NA	NA
Mobility CF	0.52 (0.23, 1.20)	0.42 (0.13, 1.37)	0.53 (0.19, 1.45)	0.48 (0.14, 1.62)	NA	NA
Mobility RCF	NA	NA	NA	NA	0.52 (0.16, 1.71)	0.39 (0.07, 2.33)
Mobility PRCF	NA	NA	NA	NA	0.39 (0.13, 1.24)	0.21 (0.03, 1.45)
Non-mobility CF	1.25 (0.34, 4.61)	0.61 (0.09, 4.25)	0.73 (0.14, 3.75)	0.57 (0.10, 3.28)	NA	NA
Non-mobility RCF	NA	NA	NA	NA	0.96 (0.19, 4.76)	NA
Non-mobility PRCF	NA	NA	NA	NA	0.72 (0.11, 4.67)	NA
Cognitive decline	0^a^	0^a^	0^a^	–	–	–
Pre-MCI	NA	NA	NA	1.01 (0.19, 5.55)	0.66 (0.18, 2.40)	0.91 (0.12, 7.07)
MCI	–	–	–	0^a^	0^a^	0^a^
BMI	1.13 (1.01, 1.25)*	–	–	–	–	–
CVD (ref: yes)	–	–	0.54 (0.26, 1.14)	–	–	–
Social dysfunction	–	–	0.95 (0.92, 0.98)**	–	–	–
GDS15	–	0.83(0.70, 0.97)*	–	–	–	–
Recognition	–	–	–	–	–	0.97 (0.62, 1.54)
Boston naming	–	–	–	–	–	0.83 (0.49, 1.41)
TMT A	–	–	1.02 (0.90, 1.15)	1.05 (0.93, 1.20)	1.01 (0.92, 1.10)	–
	Tinnitus (1, ref: 2)					
**Frailty phenotypes**						
Robust	NA	0.20 (0.03, 1.54)	0.16 (0.02, 1.12)			0.10 (0.01, 1.48)
Mobility frailty	NA		0.53 (0.06, 4.54)	0.40 (0.04, 3.99)		0.19 (0.01, 3.46)
Non-mobility frailty	NA		0.42 (0.03, 6.92)	0.28 (0.02, 4.78)		
Mobility CF	0.36 (0.12, 1.07)	0.24 (0.05, 1.23)	0.26 (0.06, 1.14)	0.20 (0.04, 1.14)		
Mobility RCF	NA	NA	NA	NA	0.21 (0.04, 1.16)	0.15 (0.01, 2.15)
Mobility PRCF	NA	NA	NA	NA	0.35 (0.07, 1.65)	1.26 (0.07, 21.83)
Non-mobility CF	1.09 (0.21, 5.60)	0.48 (0.03, 7.22)	1.31 (0.17, 9.89)	0.92 (0.11, 7.94)	NA	NA
Non-mobility RCF	NA	NA	NA	NA	0.47 (0.05, 4.33)	NA
Non-mobility PRCF	NA	NA	NA	NA	1.01 (0.10, 10.46)	NA
Cognitive decline	0^a^	0^a^	0^a^	–	–	–
Only pre-MCI	NA	NA	NA	0.70 (0.08, 5.96)	0.54 (0.10, 2.83)	2.58 (0.15, 45.30)
MCI	–	–	–	0^a^	0^a^	0^a^
BMI	1.17 (1.01, 1.35)*	–	–	–	–	–
CVD (ref: yes)	–	–	0.19 (0.46, 0.75)*	–	–	–
Social dysfunction	–	–	0.96 (0.91, 1.02)	–	–	–
GDS15	–	0.95 (0.74, 1.22)	–	–	–	–
Recognition	–	–	–	–	–	3.74 (1.15, 12.18)*
Boston naming	–	–	–	–	–	2.88 (0.99, 8.38)
TMT A	–	–	0.75 (0.60, 0.93)**	0.75 (0.59, 0.94)*	0.80 (0.67, 0.96)*	–

*p < 0.05 and **p ≤ 0.01. ^*a*^means reference category; OR = odds ratios; CI = confidence intervals; NA, not applicable; CF = cognitive frailty; CVD, cardiovascular disease; RCF = reversible cognitive frailty; PRCF = potential reversible cognitive frailty; MCI, mild cognitive impairment; BMI, body mass index; TMT A, Trail Making Test A; GDS, the Geriatric Depression Scale; BNT, Boston naming. Model 1: adjusted for mobility CF, non-mobility CF, cognitive decline, and BMI; Model 2: adjusted for robust, mobility CF, non-mobility CF, cognitive decline, and GDS15; Model 3: adjusted for robust, mobility frailty; non-mobility frailty; mobility CF, non-mobility CF, cognitive decline, CVD, social dysfunction, and TMT A. Model 4: adjusted for mobility frailty; non-mobility frailty; mobility CF, non-mobility CF, pre-MCI, MCI, and TMT A; Model 5: adjusted for mobility frailty; mobility RCF, mobility PRCF, non-mobility RCF, non-mobility PRCF, pre-MCI, MCI, and TMT A. Model 6 is adjusted for robust, mobility frailty; mobility RCF, mobility PRCF, non-mobility RCF, non-mobility PRCF, pre-MCI, MCI, recognition, and BNT. Tinnitus 0 = no tinnitus; tinnitus 1 = mild or moderate tinnitus; tinnitus 2 = severe or disastrous tinnitus.

Among the six frailty stratifications in model 3 of Table 4, robust (OR, 4.23) and mobility frailty (OR, 11.43) phenotypes were associated with significantly higher odds of without HL and tinnitus, or with tinnitus (OR, 9.81) than those of HL and tinnitus when compared with the cognitive decline group. The mobility frailty phenotype was associated with significantly higher odds of without HL and tinnitus (OR, 36.41) and only tinnitus (OR, 7.92) than those of HL and tinnitus when compared with the MCI group (model 4, Table 4). The non-mobility RCF (OR, 5.29; *p* = 0.055) and pre-MCI (OR, 3.97; *p* = 0.067) stratifications were associated with marginally higher odds of without HL and tinnitus than those of HL and tinnitus when compared with the MCI stratification (model 5, Table 4). After excluding the non-mobility frailty phenotypes, the robust (OR, 8.73; *p* = 0.092) and mobility frailty (OR, 25.31) phenotypes were associated with higher odds of without HL and tinnitus than those of HL and tinnitus (model 6, Table 4). Age was an independent confounder associated with the presentation of HL and/or tinnitus (*p* = 0.054 in model 2; *p* = 0.065 in model 3; *p* = 0.078 in model 6; and *p* < 0.05 in other models; Table 4). Social dysfunction (model 1, Table 4), BNT score (models 2 and 3), and CVD (model 3, Table 4) were also independent confounders associated with the presentation of HL and/or tinnitus.

**TABLE 4:** Association between frailty phenotype and comorbid hearing loss and tinnitus by using multivariate logistic regression or nominal regression.

	**HL and tinnitus (0, ref: 3)**					
	**Model 1 OR (95%CI)**	**Model 2 OR (95%CI)**	**Model 3 OR (95%CI)**	**Model 4 OR (95%CI)**	**Model 5 OR (95%CI)**	**Model 6 OR (95%CI)**
**Frailty phenotypes**						
Robust	NA	3.23 (0.72, 14.59)	4.23 (1.06, 16.97)*	NA	NA	**8.73 (0.71, 108.25)**
Mobility frailty	NA	NA	11.43 (2.14, 61.11)**	36.41 (3.10, 428.33)**	NA	25.31 (1.68, 380.46)*
Non-mobility frailty	NA	NA	2.19 (0.26, 18.32)	7.27 (0.44, 119.28)	NA	NA
Mobility CF	0.56 (0.20, 1.62)	0.86 (0.19, 3.79)	1.14 (0.31, 4.19)	4.34 (0.46, 41.14)	NA	NA
Mobility RCF	NA	NA	NA	NA	1.89 (0.43, 8.40)	2.60 (0.19, 34.95)
Mobility PRCF	NA	NA	NA	NA	1.14 (0.25, 5.12)	1.25 (0.08, 18.99)
Non-mobility CF	2.10 (0.54, 8.14)	1.11 (0.10, 11.76)	1.91 (0.29, 12.43)	6.21 (0.45, 85.46)	NA	NA
Non-mobility RCF	NA	NA	NA	NA	**5.29 (0.97, 28.99)**	NA
Non-mobility PRCF	NA	NA	NA	NA	3.23 (0.36, 29.00)	NA
Cognitive decline	0^a^	0^a^	0^a^	–	–	–
Pre-MCI	NA	NA	NA	7.88 (0.66, 94.19)	**3.97(0.91, 17.32)**	3.75 (0.26, 54.09)
MCI	–	–	–	0^a^	0^a^	0^a^
Age	0.86 (0.80, 0.93)***	0.82 (0.74, 0.91)***	0.86 (0.80, 0.93)***	0.85 (0.78, 0.93)***	0.87 (0.81, 0.94)***	0.83 (0.76, 0.91)***
CVD (ref: yes)	–	–	1.13 (0.46, 2.79)	–	–	–
Social dysfunction	0.97 (0.92, 1.02)	–	–	–	–	–
BNT	–	0.66 (0.32, 1.35)	0.82 (0.48, 1.38)	–	–	0.55 (0.26, 1.17)
	HL and tinnitus (1, ref: 3)					
**Frailty phenotypes**						
Robust	NA	1.41 (0.37, 5.45)	1.39 (0.42, 4.59)	NA	NA	1.56 (0.27, 8.82)
Mobility frailty	NA	NA	1.99 (0.42, 9.46)	1.20 (0.25, 5.84)	NA	2.48 (0.32, 19.05)
Non-mobility frailty	NA	NA	0.74 (0.11, 5.25)	0.60 (0.08, 4.36)	NA	NA
Mobility CF	0.93 (0.40, 2.19)	0.55 (0.17, 1.85)	0.56 (0.22, 1.46)	0.65 (0.23, 1.82)	NA	NA
Mobility RCF	NA	NA	NA	NA	0.65 (0.22, 1.97)	0.53 (0.09, 3.15)
Mobility PRCF	NA	NA	NA	NA	0.83 (0.30, 2.32)	0.56 (0.10, 3.23)
Non-mobility CF	0.58 (0.14, 2.34)	0.42 (0.04, 5.00)	0.46 (0.8, 2.83)	0.37 (0.06, 2.36)	NA	NA
Non-mobility RCF	NA	NA	NA	NA	0.34 (0.06, 2.02)	NA
Non-mobility PRCF	NA	NA	NA	NA	1.18 (0.20, 7.11)	NA
Cognitive decline	0^a^	0^a^	0^a^	–	–	–
Only pre-MCI	NA	NA	NA	0.89 (0.20, 4.02)	0.92 (0.26, 3.28)	1.08 (0.16, 7.24)
MCI	–	–	–	0^a^	0^a^	0^a^
Age	0.99 (0.93, 1.06)	0.95 (0.88, 1.03)	0.97 (0.92, 1.03)	0.98 (0.92, 1.04)	1.00 (0.95, 1.06)	0.95 (0.88, 1.03)
CVD (ref: yes)	–	–	0.59 (0.26, 1.36)	–	–	–
Social dysfunction	0.94 (0.90, 0.98)**	–	–	–	–	–
BNT	–	0.46 (0.26, 0.83)**	0.57 (0.38, 0.87)**	–	–	0.43 (0.22, 0.82)*
	HL and tinnitus (2, ref: 3)					
**Frailty phenotypes**						
Robust	NA	1.91 (0.37, 9.91)	3.75 (0.77, 18.36)	NA	NA	2.07 (0.25, 17.16)
Mobility frailty	NA	NA	9.81 (1.54, 62.34)*	7.92 (1.02, 61.77)*	NA	5.57 (0.53, 58.21)
Non-mobility frailty	NA	NA	2.80 (0.32, 24.67)	3.30 (0.31, 35.32)	NA	NA
Mobility CF	0.65 (0.20, 2.10)	0.66 (0.14, 3.27)	0.92 (0.21, 4.08)	1.17 (0.20, 6.99)	NA	NA
Mobility RCF	NA	NA	NA	NA	2.82 (0.58, 13.73)	1.29 (0.16, 10.53)
Mobility PRCF	NA	NA	NA	NA	0.25 (0.02, 2.76)	NS
Non-mobility CF	0.89 (0.14, 5.52)	1.81 (0.19, 16.99)	1.74 (0.22, 13.53)	2.11 (0.22, 20.39)	NA	NA
Non-mobility RCF	NA	NA	NA	NA	2.30 (0.28, 19.04)	NA
Non-mobility PRCF	NA	NA	NA	NA	NS	NA
Cognitive decline	0^a^	0^a^	0^a^	–	–	–
Only pre-MCI	NA	NA	NA	1.78 (0.19, 17.13)	3.54 (0.70, 17.95)	0.93 (0.08, 10.67)
MCI	–	–	–	0^a^	0^a^	0^a^
Age	0.87 (0.80, 0.95)**	**0.90 (0.82, 1.00)**	0.90 (0.83, 0.98)*	**0.92 (0.84, 1.01)**	0.88 (0.80, 0.96)**	**0.91 (0.83, 1.01)**
CVD (ref: yes)	–	–	0.25 (0.07, 0.87)*	–	–	–
Social dysfunction	0.92 (0.86, 0.99)*	–	–	–	–	–
BNT	–	0.70 (0.33, 1.50)	0.67 (0.39, 1.15)	–	–	0.55 (0.24, 1.22)

*p < 0.05; **p ≤ 0.01; ***p < 0.001; bold values denote marginally statistical significance. a means reference category; OR = odds ratios; CI = confidence intervals; CVD, cardiovascular disease; NA, not applicable; NS, no significance; RCF = reversible cognitive frailty; PRCF = potential reversible cognitive frailty; MCI, mild cognitive impairment; HL, hearing loss; BNT, Boston naming. Model 1 is adjusted for mobility CF, non-mobility CF, cognitive decline, age, and social dysfunction. Model 2 is adjusted for robust, mobility CF, non-mobility CF, cognitive decline, age, and BNT. Model 3 is adjusted for robust, mobility frailty; non-mobility frailty; mobility CF, non-mobility CF, cognitive decline, age, CVD, and BNT. Model 4 is adjusted for mobility frailty; non-mobility frailty; mobility CF, non-mobility CF, pre-MCI, MCI, and age. Model 5 is adjusted for mobility RCF, mobility PRCF, non-mobility RCF, non-mobility PRCF, pre-MCI, MCI, and age. Model 6 is adjusted for robust, mobility frailty; mobility RCF, mobility PRCF, pre-MCI, MCI, age, and BNT. HL and tinnitus 0 = without HL and tinnitus; HL and tinnitus 1 = only HL; HL and tinnitus 0 = only tinnitus; HL and tinnitus 3 = with HL and tinnitus.

## Discussion

From the cross-sectional study, we found that frailty phenotypes and CF subtypes had a different association with the severity of HL and tinnitus and presented HL and/or tinnitus. Patients with physical frailty, such as mobility frailty or who are robust had lower probability with severe HL, tinnitus, and presented HL and/or tinnitus than those with cognitive decline, CF, and mobility RCF and PRCF. Patients with RCF and non-mobility RCF had higher probability with less HL, tinnitus, and presented HL and/or tinnitus than those with PRCF and mobility RCF. Our findings provided additional evidence supporting the results of a previous longitudinal study, which indicated that the frailty phenotypes are heterogeneous with different longitudinal trajectories of mortality and disability (Romero-Ortuno et al., 2019).

Although many epidemiological studies indicated that physical frailty increases the risk of future cognitive decline (Robertson et al., 2013), the addition of cognitive impairment to the assessment of physical frailty may improve the prediction of adverse outcomes of physical frailty during the later stages of life (Lee et al., 2018), including death from heart transplantation (Jha et al., 2016), death among oldest-old individuals (Brigola et al., 2020), functional decline, falls, and hospitalization (Hao et al., 2018). The overall or individual domain score for cognitive decline in the Chinese version of the mini-mental state examination may improve the pre-frailty predictive power for poor quality of life, incident physical limitation, increased cumulative hospital stay, and mortality (Yu et al., 2018). Our study indicated that individuals with cognitive decline or CF had higher risks of severe HL and tinnitus and presented with HL and/or tinnitus. Moreover, individuals with RCF had lower risks of severe HL and tinnitus and presented with HL and/or tinnitus. Similarly, individuals with non-mobility RCF had lower risks of severe HL and tinnitus than those with mobility RCF. Slowness has been reported as the most related physical component to cognitive impairment (Mielke et al., 2013; Chhetri et al., 2017) and health-related quality of life (Henchoz et al., 2017). Indeed, motor cognitive risk syndrome has been considered as an important disease (Cohen et al., 2016; Chhetri et al., 2017). Our results extend the significant association between physical frailty, CF, CF subtype, and RCF phenotype and adverse health outcomes of older adults. These results support the evidence that CF may be an important clinical syndrome with physical and cognitive heterogeneities. Motor cognitive risk syndrome may be defined as a phenotype of CF.

Apart from frailty phenotypes, age was the most significant independent risk factor for HL severity and HL with tinnitus. Previous epidemiological studies revealed that the prevalence of sensory and motor dysfunction and cognition deficit, frailty, and tinnitus increases with age (Shargorodsky et al., 2010; Panza et al., 2015; Ruan et al., 2018; Jafari et al., 2019). Although HL is associated with cognitive impairment, frailty, and motor dysfunction (Chen et al., 2014; Panza et al., 2015; Kamil et al., 2016; Deal et al., 2017; Bang et al., 2020; Bonfiglio et al., 2020), identifying the causal relationship between HL and frailty phenotype and cognition decline is difficult because HL is similar to pre-MCI, with long subclinical period (Golub et al., 2019, 2020). Our results revealed that aging does not increase the severity of tinnitus and confirmed the reports of previous studies, which indicated that depression increases the risk for severe HL or tinnitus (Shargorodsky et al., 2010; Langguth et al., 2013; House et al., 2018; Ruan et al., 2018; Jafari et al., 2019; Golub et al., 2020). Hypertension was a risk factor for tinnitus (Shargorodsky et al., 2010). The present study demonstrated that patients with CVD have a higher risk for severe and disastrous tinnitus and HL with tinnitus than tinnitus only. Among patients with cognitive decline, BMI was independently associated with the severity of tinnitus. This finding indicates that obesity and metabolic diseases can affect the severity of tinnitus. In fact, the cardiometabolic risk factors, such as hypertension and waist circumference, had a weak correlation with tinnitus and THI level (Langguth et al., 2013; House et al., 2018). Although sex is an important risk factor for cognitive decline, and female older people have high risk for cognitive impairment and frailty (Ruan et al., 2017), our results did not show sexual difference in frailty phenotypes, HL, tinnitus, motor dysfunction, and cognitive decline.

Cognitive function is associated with HL and tinnitus. Individuals with HL had memory (Deal et al., 2017) and executive dysfunctions (Lin et al., 2013). The cognitive deficits of patients with tinnitus included executive domain, working memory, processing speeds, and attention (Ruan et al., 2018; Jafari et al., 2019). Our study further confirmed that the differences in HL and tinnitus severity and the presentation of HL and/or tinnitus are independently associated with the z-scores of memory (recognition), attention, and executive function (TMT A and TMT B), as well as language (BNT and animal fluency). Patients with attention and executive (TMT A and/or B) dysfunctions had a higher risk for more severe HL and tinnitus among the frailty phenotype and CF subtype stratifications. Patients with language domain (BNT) dysfunction had a higher risk for the presentation of HL with tinnitus rather than HL alone. Although cognition was another independent risk factor for the severity of HL, severity of tinnitus and the presentation of HL and/or tinnitus, the causal relationship between HL or tinnitus and cognition remains elusive.

The total amount of social dysfunction might be detected with the Social Dysfunction Rating Scale optimal cut-off value ≥ 26 (Lozupone et al., 2018). The cut-off value could be used to detect social vulnerabilities, including social frailty. Social dysfunction or social frailty has been validated to be associated with cognitive, depression and HL (Lozupone et al., 2018; Ma et al., 2018; Yoo et al., 2019), and the relationship between cognition and hearing loss (Loughrey et al., 2020). In this study, social dysfunction was also an independent risk factor for the differences in the severity of HL, tinnitus, and presented HL and/or tinnitus. Individuals with severe social dysfunction had a higher risk for severe tinnitus and presented HL and tinnitus rather than HL alone or tinnitus alone. Social dysfunction or isolation and loneliness due to communication impairment had been linked to HL and cognitive deficits (Panza et al., 2015). Social factors may influence tinnitus perception, interpretation, and mental representation and were considered in patients with tinnitus (Li et al., 2015). The potential link mechanism of social dysfunction and tinnitus and presentation of HL with tinnitus need further investigation.

Multidisciplinary studies showed that peripheral and central HL, and motor dysfunction are observed in the preclinical AD stage. The major AD pathological changes, including amyloid plaques and neurofibrillary tangles, were observed in the central auditory neural pathway, primary motor cortex, and supplementary motor areas in AD patients. Interventions targeting the amelioration of sensory-motor deficits in AD may enhance patient function as AD progresses (Albers et al., 2015). The common-cause hypothesis that systematic age-related nervous system pathologies such as AD pathology, brain atrophy, and reduced dendritic spine densities in widespread brain regions are linked to HL, tinnitus, and dementia risks had been supported by many studies (Panza et al., 2015; Jafari et al., 2019). The common etiological pathways, including microvascular disease, inflammation, metabolic dysfunction, and nutritional and hormonal factors, lead to HL, tinnitus, motor impairment, and cognitive decline. Social dysfunction or frailty is the immediate stage between HL and/or tinnitus and cognitive decline (Panza et al., 2015). Our results show that patients with a frailty phenotype that involves cognitive or mobility decline had higher risks of severe HL and tinnitus, and presented with HL and/or tinnitus. These results also provide additional evidence to the common-cause hypothesis.

A major strength of this study is the objective cognitive measures. Cognitive status was measured on the basis of the normative *z*-scores of six neuropsychological tests and process *z*-scores of HVLT-R (Ruan et al., 2020b), which decrease the diagnostic errors resulting from the clinical evaluation for MCI or subjective questionnaire for pre-MCI. The present study shows that implementation of integrated care based on intrinsic capacity (World Health Organization, 2017), including sensory, motor, cognitive performance, and frailty status of older people, is necessary in clinical practice. Although this cohort includes a sample representing community-dwelling older adults and has a substantial number of potential confounders, one main limitation in this study is the sample size. The number of patients in the non-mobility CF stratification, especially the non-mobility CF subtypes, RCF and PRCF stratifications, are small, which limits the representativeness of the population, and the conclusion about the differences in their association with HL, tinnitus, and HL with tinnitus. In addition, the cross-sectional study cannot determine the causal relationship between independent risk factors and HL, tinnitus, and HL with tinnitus. Finally, although social dysfunction and depression were validated to be independent confounders of the severity of HL and tinnitus, this study focused on mobility and non-mobility frailty, mobility and non-mobility CF and their subtypes, and other dimensions such as social and psychological frailty phenotypes (Lozupone et al., 2018; Ma et al., 2018; Solfrizzi et al., 2019; Yoo et al., 2019), and loneliness as a study variable assessed by using a validated scale, which are related to the severity of HL and tinnitus, and presentations of HL and tinnitus. These require further investigation in a future study. Future research should further explore the relationship between multi-sensory dysfunction, cognitive decline, and frailty phenotypes to develop person-centered assessment, and integrated care in clinical practice.

Frailty phenotypes had different associations with the severity of HL and tinnitus, and the presentation of HL with tinnitus. Patients with cognitive decline or CF had higher risk for severe HL and tinnitus, and presented HL with tinnitus than robust and those with physical frailty. Patients with RCF or non-mobility RCF had lower risk for severe HL and tinnitus, and presented HL with tinnitus than those with PRCF or mobility RCF.

## Data Availability Statement

The original contributions presented in the study are included in the article/supplementary material, further inquiries can be directed to the corresponding author/s.

## Ethics Statement

The study was approved by the Ethics Committee of Huadong hospital and written informed consent was obtained from each volunteer or authorized representative. The patients/participants provided their written informed consent to participate in this study.

## Author Contributions

QR and ZY designed the study and drafted the initial version of the manuscript. QR, JC, WZ, JR, and ZY collected the data, performed clinical and neuropsychological test measures, and data analysis. MZ, RZ, and CH performed hearing and tinnitus assessment. All authors contributed to the final version of the manuscript.

## Conflict of Interest

The authors declare that the research was conducted in the absence of any commercial or financial relationships that could be construed as a potential conflict of interest.

## References

[B1] Albers M. W. Gilmore G. C. Kaye J. Murphy C. Wingfield A. Bennett D. A. (2015). At the interface of sensory and motor dysfunctions and Alzheimer’s disease. *Alzheimers Dement.* 11 70–98. 25022540 10.1016/j.jalz.2014.04.514 PMC4287457

[B2] American Psychiatric Association (1994). *Diagnostic and Statistical Manual of Mental Disorders*, 4th Edn. Washington, DC: Americian Psychiartric Association.

[B3] Andrew M. K. Mitnitski A. B. Rockwood K. (2008). Social vulnerability, frailty and mortality in elderly people. *PLoS One* 3:e2232. 10.1371/journal.pone.0002232 18493324 PMC2375054

[B4] Bae S. Lee S. Lee S. Harada K. Makizako H. Park H. (2018). Combined effect of self-reported hearing problems and level of social activities on the risk of disability in Japanese older adults: a population-based longitudinal study. *Maturitas* 115 51–55. 10.1016/j.maturitas.2018.06.008 30049347

[B5] Bang S. H. Jeon J. M. Lee J. G. Choi J. Song J. J. Chae S. W. (2020). Association between hearing loss and postural instability in older Korean adults. *JAMA Otolaryngol. Head Neck Surg.* 146 530–534. 10.1001/jamaoto.2020.0293 32324231 PMC7180733

[B6] Bonfiglio V. Umegaki H. Kuzuya M. (2020). A study on the relationship between cognitive performance, hearing impairment, and frailty in older adults. *Dement. Geriatr. Cogn. Disord.* 49 156–162. 10.1159/000507214 32320988

[B7] Boyle P. A. Buchman A. S. Wilson R. S. Leurgans S. E. Bennett D. A. (2009). Association of muscle strength with the risk of Alzheimer disease and the rate of cognitive decline in community-dwelling older persons. *Arch. Neurol.* 66 1339–1344. 19901164 10.1001/archneurol.2009.240 PMC2838435

[B8] Brigola A. G. Ottaviani A. C. Alexandre T. D. S. Luchesi B. M. Pavarini S. C. I. (2020). Cumulative effects of cognitive impairment and frailty on functional decline, falls and hospitalization: a four-year follow-up study with older adults. *Arch. Gerontol. Geriatr.* 87:104005. 10.1016/j.archger.2019.104005 31901850

[B9] Bunt S. Steverink N. Olthof J. van der Schans C. P. Hobbelen J. S. M. (2017). Social frailty in older adults: a scoping review. *Eur. J. Ageing.* 31 323–334. 10.1007/s10433-017-0414-7 28936141 PMC5587459

[B10] Carpenter-Thompson J. R. McAuley E. Husain F. T. (2015). Physical activity, tinnitus severity, and improved quality of life. *Ear Hear.* 36 574–581. 10.1097/aud.0000000000000169 25906172

[B11] Chau J. Martin C. R. Thompson D. R. Chang A. M. Woo J. (2006). Factor structure of the Chinese version of the geriatric depression scale. *Psychol. Health Med.* 11 48–59. 10.1080/13548500500093688 17129894

[B12] Chen D. S. Genther D. J. Betz J. Lin F. R. (2014). Association between hearing impairment and self-reported difficulty in physical functioning. *J. Am. Geriatr Soc.* 62 850–856. 10.1111/jgs.12800 24779559 PMC4084895

[B13] Chhetri J. K. Chan P. Vellas B. Cesari M. (2017). Motoric cognitive risk syndrome: predictor of dementia and age-related negative outcomes. *Front. Med.* 4:166. 10.3389/fmed.2017.00166 29119100 PMC5660976

[B14] Cohen J. A. Verghese J. Zwerling J. L. (2016). Cognition and gait in older people. *Maturitas* 93 73–77. 10.1016/j.maturitas.2016.05.005 27240713

[B15] Deal J. A. Betz J. Yaffe K. Harris T. Purchase-Helzner E. Satterfield S. (2017). Hearing impairment and incident dementia and cognitive decline in older adults: the health ABC Study. *J. Gerontol. A Biol. Sci. Med. Sci.* 72 703–709. 27071780 10.1093/gerona/glw069 PMC5964742

[B16] Fried L. P. Tangen C. M. Walston J. Newman A. B. Hirsch C. Gottdiener J. (2001). Frailty in older adults: evidence for a phenotype. *J. Gerontol. A Biol. Sci. Med. Sci.* 56 M146–M156. 11253156 10.1093/gerona/56.3.m146

[B17] Golub J. S. Brewster K. K. Brickman A. M. Ciarleglio A. J. Kim A. H. Luchsinger J. A. (2020). Subclinical hearing loss is associated with depressive symptoms. *Am. J. Geriatr. Psychiatry* 28 545–556. 10.1016/j.jagp.2019.12.008 31980375 PMC7324246

[B18] Golub J. S. Brickman A. M. Ciarleglio A. J. Schupf N. Luchsinger J. A. (2019). Association of subclinical hearing loss with cognitive performance. *JAMA Otolaryngol. Head Neck Surg.* 146 57–67. 10.1001/jamaoto.2019.3375 31725853 PMC6865840

[B19] Hao Q. Dong B. Yang M. Dong B. Wei Y. (2018). Frailty and cognitive impairment in predicting mortality among oldest-old people. *Front. Aging Neurosci.* 10:295. 10.3389/fnagi.2018.00295 30405390 PMC6201058

[B20] Hao Q. K. Li J. Dong B. R. (2017). Chinese experts consensus on assessment and intervention for elderly patients with frailty. *Chin. J. Geriatr.* 36 251–256.

[B21] HenchozY.BülaC.GuessousI.Santos-EggimannB. (2017). Association between physical frailty and quality of life in a representative sample of community-dwelling Swiss older people. *J. Nutr. Health Aging.* 21 585–592. 10.1007/s12603-016-0772-4 28448091

[B22] Hoogendijk E. O. Afilalo J. Ensrud K. E. Kowal P. Onder G. Fried L. P. (2019). Frailty: implications for clinical practice and public health. *Lancet* 394 1365–1375. 10.1016/s0140-6736(19)31786-6 31609228

[B23] House L. Bishop C. E. Spankovich C. Su D. Valle K. Schweinfurth J. (2018). Tinnitus and its risk factors in African Americans: the Jackson heart study. *Laryngoscope* 128 1668–1675. 10.1002/lary.26964 29193110 PMC5975087

[B24] Jafari Z. Kolb B. E. Mohajerani M. H. (2019). Age-related hearing loss and tinnitus, dementia risk, and auditory amplification outcomes. *Ageing Res. Rev.* 56:100963. 10.1016/j.arr.2019.100963 31557539

[B25] Jha S. R. Hannu M. K. Gore K. Chang S. Newton P. Wilhelm K. (2016). Cognitive impairment improves the predictive validity of physical frailty for mortality in patients with advanced heart failure referred for heart transplantation. *J. Heart. Lung. Transplant.* 35 1092–1100. 10.1016/j.healun.2016.04.008 27282417

[B26] Kamil R. J. Betz J. Powers B. B. Pratt S. Kritchevsky S. Ayonayon H. N. (2016). Association of hearing impairment with incident frailty and falls in older adults. *J. Aging Health*. 28 644–660. 10.1177/0898264315608730 26438083 PMC5644033

[B27] KelaiditiE.CesariM.CanevelliM.van KanG. A.OussetP. J.Gillette-GuyonnetS. (2013). Cognitive frailty: rational and definition from an (I.A.N.A/I.A.G.G.) international consensus group. *J. Nutr. Health Aging* 17 726–734. 10.1007/s12603-013-0367-2 24154642

[B28] Langguth B. Kreuzer P. M. Kleinjung T. De Ridder D. (2013). Tinnitus: causes and clinical management. *Lancet Neurol.* 12 920–930. 10.1016/s1474-4422(13)70160-1 23948178

[B29] Lee Y. Kim J. Chon D. Lee K. E. Kim J. H. Myeong S. (2018). The effects of frailty and cognitive impairment on 3-year mortality in older adults. *Maturitas* 107 50–55. 10.1016/j.maturitas.2017.10.006 29169580

[B30] Li Z. Gu R. Zeng X. (2015). The social-neurophysiological model of tinnitus: theory and practice. *J. Formos. Med. Assoc.* 114 201–203. 10.1016/j.jfma.2013.09.003 24083913

[B31] Lin F. R. Yaffe K. Xia J. Xue Q. L. Harris T. B. Purchase-Helznery E. (2013). Hearing loss and cognitive decline in older adults. *JAMA Intern. Med.* 173, 293–299. 10.1001/jamainternmed.2013.1868 23337978 PMC3869227

[B32] Linn M. W. Sculthorpe W. B. Evje M. Slater P. H. Goodman S. P. (1969). A social dysfunction rating scale. *J. Psychiatr. Res.* 6 299–306. 5789952 10.1016/0022-3956(69)90023-5

[B33] Liu L. K. Guo C. Y. Lee W. J. Chen L. Y. Hwang A. C. Lin M. H. (2017). Subtypes of physical frailty: latent class analysis and associations with clinical characteristics and outcomes. *Sci. Rep.* 7:46417. 10.1038/srep46417 PMC5387710 28397814

[B34] Loughrey D. G. Feeney J. Kee F. Lawlor B. A. Woodside J. V. Setti A. (2020). Social factors may mediate the relationship between subjective age-related hearing loss and episodic memory. *Aging Ment. Health.* 18 1–8. 10.1080/13607863.2020.1727847 32067488

[B35] Lozupone M. Panza F. Piccininni M. Copetti M. Sardone R. Imbimbo B. P. (2018). Social dysfunction in older age and relationships with cognition, depression, and apathy: the GreatAGE study. *J. Alzheimers Dis.* 65 989–1000. 10.3233/jad-180466 30103335

[B36] MaL.SunF.TangZ. (2018). Social frailty is associated with physical functioning, cognition, and depression, and predicts mortality. *J. Nutr. Health Aging.* 22 989–995. 10.1007/s12603-018-1054-0 30272104

[B37] Maharani A. Pendleton N. Leroi I. (2019). Hearing impairment, loneliness, social isolation, and cognitive function: longitudinal analysis using English longitudinal study on ageing. *Am. J. Geriatr. Psychiatry.* 27 1348–1356. 10.1016/j.jagp.2019.07.010 31402088

[B38] Mielke M. M. Roberts R. O. Savica R. Cha R. Drubach D. I. Christianson T. (2013). Assessing the temporal relationship between cognition and gait: slow gait predicts cognitive decline in the Mayo Clinic Study of Aging. *J. Gerontol. A Biol. Sci. Med. Sci.* 68 929–937. 10.1093/gerona/gls256 23250002 PMC3712358

[B39] Newman C. W. Jacobson G. P. Spitzer J. B. (1996). Development of the tinnitus handicap inventory. *Arch. Otolaryngol. Head Neck Surg.* 122 143–148. 8630207 10.1001/archotol.1996.01890140029007

[B40] Panza F. Solfrizzi V. Seripa D. Imbimbo B. P. Capozzo R. Quaranta N. (2015). Age-related hearing impairment and frailty in Alzheimer’s disease: interconnected associations and mechanisms. *Front. Aging Neurosci.* 7:113. 10.3389/fnagi.2015.00113 26106327 PMC4460423

[B41] Robertson D. A. Savva G. M. Kenny R. A. (2013). Frailty and cognitive impairment–a review of the evidence and causal mechanisms. *Ageing Res Rev.* 12 840–851. 10.1016/j.arr.2013.06.004 23831959

[B42] Romero-Ortuno R. Scarlett S. O’Halloran A. M. Kenny R. A. (2019). Is phenotypical prefrailty all the same? A longitudinal investigation of two prefrailty subtypes in TILDA. *Age Ageing.* 49 39–45. 10.1093/ageing/afz129 31711148

[B43] Ruan Q. D’onofrio G. Wu T. Greco A. Sancarlo D. Yu Z. (2017). Sexual dimorphism of frailty and cognitive impairment: potential underlying mechanisms. *Mol. Med. Rep.* 16 3023–3033. 10.3892/mmr.2017.6988 28713963

[B44] RuanQ.HuangY.YangL.LiJ.GuW.BaoZ. (2020a). Associations of preoperative irisin levels of paired cerebrospinal fluid and plasma with physical dysfunction and muscle wasting severity in residents of surgery wards. *J. Nutr. Health Aging.* 24 412–422. 10.1007/s12603-020-1343-232242209

[B45] Ruan Q. Xiao F. Gong K. Zhang W. Zhang M. Ruan J. (2020b). Demographically corrected normative Z-scores on the neuropsychological test battery in cognitively normal older Chinese adults. *Dem. Geriatr. Cogn. Disord.* 10.1159/000505618 Online ahead of print 33176318

[B46] RuanQ.XiaoF.GongK.ZhangW.ZhangM.RuanJ. (2020c). Prevalence of cognitive frailty phenotypes and associated factors in a community-dwelling elderly population. *J. Nutr. Health Aging.* 24 172–180. 10.1007/s12603-019-1286-7 32003407

[B47] Ruan Q. Yu Z. Chen M. Bao Z. Li J. He W. (2015). Cognitive frailty, a novel target for the prevention of elderly dependency. *Ageing Res. Rev.* 20 1–10. 10.1016/j.arr.2014.12.004 25555677

[B48] Ruan Q. Yu Z. Zhang W. Ruan J. Liu C. Zhang R. (2018). Cholinergic hypofunction in presbycusis-related tinnitus with cognitive function impairment: emerging hypotheses. *Front. Aging Neurosci.* 10:98. 10.3389/fnagi.2018.00098 29681847 PMC5897739

[B49] Shargorodsky J. Curhan G. C. Farwell W. R. (2010). Prevalence and characteristics of tinnitus among US adults. *Am. J. Med.* 123 711–718. 10.1016/j.amjmed.2010.02.015 20670725

[B50] Solfrizzi V. Scafato E. Lozupone M. Seripa D. Schilardi A. Custodero C. (2019). Biopsychosocial frailty and the risk of incident dementia: the Italian longitudinal study on aging. *Alzheimers Dement.* 15 1019–1028. 10.1016/j.jalz.2019.04.013 31278052

[B51] Thomas K. R. Bangen K. J. Weigand A. J. Edmonds E. C. Wong C. G. Cooper S. (2020). Objective subtle cognitive difficulties predict future amyloid accumulation and neurodegeneration. *Neurology* 94 e397–e406. 31888974 10.1212/WNL.0000000000008838 PMC7079691

[B52] Thomas K. R. Edmonds E. C. Eppig J. Salmon D. P. Bondi M. W. Alzheimer’s Disease Neuroimaging Initiative (2018). Using neuropsychological process scores to identify subtle cognitive decline and predict progression to mild cognitive impairment. *J. Alzheimers Dis.* 64 195–204. 10.3233/jad-180229 29865077 PMC7263028

[B53] Wallace L. M. K. Theou O. Godin J. Andrew M. K. Bennett D. A. Rockwood K. (2019). Investigation of frailty as a moderator of the relationship between neuropathology and dementia in Alzheimer’s disease: a cross-sectional analysis of data from the Rush Memory and Aging Project. *Lancet Neurol.* 18 177–184. 10.1016/s1474-4422(18)30371-5 30663607 PMC11062500

[B54] World Health Organization (2015). *Prevention of Blindness and Deafness Grades of Hearing Impairment.* Available from: http://www.who.int/pbd/deafness/hearing_impairment_grades/en/. [Accessed November 29, 2015]

[B55] World Health Organization (2017). *Integrated Care for Older People: Guidelines on Community-Level Interventions to Manage Declines in Intrinsic Capacity.* Geneva: World Health Organization. 29608259

[B56] Yoo M. Kim S. Kim B. S. Yoo J. Lee S. Jang H. C. (2019). Moderate hearing loss is related with social frailty in a community-dwelling older adults: the Korean Frailty and Aging Cohort Study (KFACS). *Arch. Gerontol. Geriatr.* 83 126–130. 10.1016/j.archger.2019.04.004 31003135

[B57] Yu R. Morley J. E. Kwok T. Leung J. Cheung O. Woo J. (2018). The effects of combinations of cognitive impairment and pre-frailty on adverse outcomes from a prospective community-based cohort study of older Chinese people. *Front. Med.* 5:50. 10.3389/fmed.2018.00050 29600247 PMC5863513

